# Combining *Bacillus* and *Trichoderma* in Bio-Organic Fertilizers with Reduced Chemical Fertilizer: An Effective Strategy Against Cucumber *Fusarium* Wilt

**DOI:** 10.3390/plants15050782

**Published:** 2026-03-04

**Authors:** Xing Luo, Jiawei Ouyang, Jing Li, Hua Yu, Song Guo, Xiangzhong Zeng, Zijun Zhou, Yuxian Shangguan, Mingjiang He, Yiting Ouyang, Kun Chen, Yusheng Qin

**Affiliations:** 1 Institute of Agricultural Resources and Environment, Sichuan Academy of Agricultural Sciences, Chengdu 610066, China; luoxingjn@scsaas.cn (X.L.); yuhua135@scsaas.cn (H.Y.); guosong@scsaas.cn (S.G.); zengxiangzhong@scsaas.cn (X.Z.); zjzhou@scsaas.cn (Z.Z.); shangguan@scsaas.cn (Y.S.); mjhe9331@scsaas.cn (M.H.); ouyangyiting@scsaas.cn (Y.O.); chenkun410@scsaas.cn (K.C.); 2 Sichuan Vegetable Engineering Technology Research Center, Chengdu 611934, China; 3 Sichuan Guojing Xingnong Investment Co., Ltd., Chengdu 610000, China; 13908181450@163.com; 4 Sichuan Institute of Edible Fungi, Chengdu 610066, China; lijingsaas@scsaas.cn

**Keywords:** cucumber *Fusarium* wilt, bio-organic fertilizers, disease management, rhizosphere community

## Abstract

Integrated fertilization using reduced chemical fertilizers and bio-organic fertilizers can maintain soil fertility with lower chemical inputs, yet its systemic effects on disease control, soil microbes, yield, and quality are not fully clear. This study aimed to: (1) evaluate the effects of *Bacillus amyloliquefaciens* Z2 and *Trichoderma harzianum* T22, alone or combined, on suppressing *Fusarium* wilt (*Fusarium oxysporum* f. sp. *cucumerinum*) and promoting cucumber growth in pot experiments; and (2) assess the field efficacy of reduced chemical fertilizer (75% N) plus microbial bio-organic fertilizer (25% N) for disease control, growth enhancement, and yield and quality improvement. To achieve these objectives, pot experiments were first conducted, followed by field experiments. Pot results indicated that individual and combined inoculants notably decreased the disease index (DI) by 40.48–68.75%, and significantly increased cucumber fresh shoot biomass by 16.86–26.75%, with the combined inoculants exhibiting the greatest effect. Field experiments indicated that the synthetic microbial bio-fertilizer has a greater advantage in promoting cucumber growth and disease suppression compared to a single bacterial bio-organic fertilizer. Specifically, the application of combined bio-fertilizers exhibited the best performance in decreasing cucumber DI by 51.54%, improving cucumber fresh shoot biomass by 12.19%, and enhancing cucumber yield by 21.02%, along with significantly improving fruit vitamin C content by 21.17% and increasing fruit total amino acids by 26.23% compared with the control. Rhizosphere soil analysis revealed that the application of combined bio-fertilizers enriched beneficial bacterial families (JG30-KF-AS9 and *Sphingomonadaceae*) and fungal genera (*Chaetomiaceae* and *Condenascus*) with known biocontrol functions and suppressed the proliferation of *Fusarium*. Overall, the integrated use of reduced chemical fertilizer combined with synthetic bio-organic fertilizer effectively suppresses cucumber wilt, optimizes microbial community structure, and improves cucumber yield and quality, furnishing a valuable foundation for microbial-assisted sustainable crop production.

## 1. Introduction

The foundation of modern intensive agriculture has long been reliant on the extensive application of chemical fertilizers to secure global food security [1]. While these inputs have boosted crop yields, their protracted and often excessive use has precipitated a severe suite of environmental and agronomic crises [1]. For example, excessive application of chemical fertilizers often leads to the degradation of soil health, such as soil compaction, acidification, and a marked decline in organic matter (OM) and biodiversity [1,2]. Furthermore, nutrient leaching and runoff also contribute significantly to environmental pollution, including eutrophication of water bodies and greenhouse gas emissions [3]. This issue is particularly acute in China’s vegetable sector, where fertilizer application rates are 3.3 times higher than those for staple crops, creating an imbalanced nutrient environment and a deteriorated soil microbiome that undermines crop resilience [4]. The above challenge is acutely manifested in the production of cucumber (*Cucumis sativus* L.), a crop of global importance due to its nutritional value, economic weight, and high consumption [5]. In addition, the practice of intensive monoculture, prevalent in facility agriculture and often accompanied by excessive chemical fertilizer use, exacerbates cucumber soil sickness, driven primarily by *Fusarium oxysporum* [6]. This pathogen invades the plant vascular system, leading to wilting, stunted growth, and yield losses of up to 80% under severe infestations [7,8]. Traditional management strategies, including chemical fungicides, crop rotation, and resistant cultivars, have shown limited efficacy. For example, excessive chemical fungicide use raises concerns about environmental contamination, pathogen resistance, and residue accumulation, and genetic resistance is often unstable due to the pathogen’s rapid evolution [9,10]. These limitations underscore the urgent need for sustainable alternatives to safeguard cucumber productivity and quality.

Biological control has emerged as a promising, eco-friendly approach to controlling crop disease [11]. Unlike chemical interventions, biocontrol agents (BCAs) could not only provide their efficacy as a green strategy to promote crop growth and manage crop diseases but also offer various advantages, such as reduced chemical fertilizers, enhanced soil health, decreased ecological disruption, and compatibility with integrated pest management systems [11,12]. Numerous studies have demonstrated the efficacy of individual BCAs against *Fusarium* wilt. For instance, Saikia et al. (2003) demonstrated that *Pseudomonas fluorescens* induced resistance against chickpea *Fusarium* wilt and notably reduced the wilt disease by 26–50% [13]; Cao et al. (2011) also reported that the application of *Bacillus velezensis* SQR 9 reduced the cucumber wilt incidence by 49–61% in greenhouse experiments [14]. Despite these successes, field-scale applications of single-strain BCAs often yield inconsistent results, attributed to environmental variability, insufficient colonization, or limited antagonistic activity under complex soil conditions [15]. This inconsistency highlights the necessity to develop advanced formulations, such as compound bio-organic fertilizers, which integrate multiple BCAs with organic substrates to enhance microbial survival, synergism, and disease suppression.

*Bacillus* spp. and *Trichoderma* spp. are prominent members of the plant growth-promoting microorganism group and are extensively used in the commercial production of organic fertilizers [16]. Specifically, *Bacillus* was reported to exhibit broad-spectrum antagonism against soil-borne pathogens via lipopeptide antibiotics production (e.g., surfactin, iturin, and fengycin) and induced systemic resistance activation [17]. For instance, *Bacillus velezensis* Bv-116 not only inhibited *F. oxysporum (Schl.)* f.sp *cucumerinum Owen* growth by 84.93% in vivo but also exhibited a significant inhibition of DI of cucumber wilt in the greenhouse pot experiment [18]. In addition, *Trichoderma* was also demonstrated to excel in mycoparasitism, secret antibiosis, and compete for nutrients or space among others to inhibit crop pathogens [19]. For instance, *Trichoderma asperellum* FJ035 not only increased the plant height, root length, stem thickness, area index of the first true leaves of cucumber seedlings, and fresh weight, but it also inhibited the cucumber *Fusarium* wilt by 75.96% and 69.79% under potted and hydroponic conditions [20]. It is worth noting that traditional in vitro studies often report antagonism between *Bacillus* and *Trichoderma*, attributed to competition for resources or chemical weapons (e.g., volatile compounds and antibiotics) cross-inhibition [21]. However, recent findings reported that double inoculation of *Bacillus* and *Trichoderma* not only enhanced the efficacy of reducing nitrate content but also exhibited the best performance in aggregate formation than that of single inoculation, finally resulting in a better cucumber yield in a secondary salinized soil [22]. In addition, Xie et al. (2024) also reported that *Bacillus* SQR9 could colonize the hyphae of *Trichoderma*, finally resulting in a notable increase in tomato growth under the co-inoculation of *Bacillus* and *Trichoderma* [23]. Hence, the capacity and critical knowledge gaps of combined bio-fertilizers containing *Bacillus* and *Trichoderma* on crop disease suppression persist. First, the efficacy of combined bio-fertilizers against *Fusarium* wilt in cucumbers remains unexplored, particularly under reduced chemical fertilization. Second, the mechanisms underlying their interactions—whether competitive, neutral, or synergistic—in shaping soil microbiomes and pathogen dynamics are poorly understood. Third, the long-term impacts of combined bio-fertilizers on crop yield and fruit quality require systematic validation under reduced chemical fertilization.

In our laboratory’s previous study, field trials were conducted to evaluate the effects of replacing chemical fertilizer nitrogen with organic fertilizer at different substitution rates, and the results indicated that the combined application of 75% chemical fertilizer nitrogen and 25% organic fertilizer nitrogen significantly enhanced cucumber yield, improved nitrogen use efficiency, increased nutritional quality (e.g., vitamin C and amino acid content), and effectively reduced nitrate levels [24]. *Bacillus amyloliquefaciens* strain Z-2, isolated from cucumber rhizosphere soil in a disease-free field and protected under Chinese invention patent ZL [CN112680377A], exhibits potent antagonistic activity against common pathogens responsible for root rot in fruits and vegetables, effectively controlling the disease. *Trichoderma harzianum* T22, a well-established beneficial microorganism commercialized in the United States, has also demonstrated notable efficacy in managing both crop diseases and pests [25]. Hence, the above two beneficial microorganisms were selected, and this study further investigated the efficacy of bio-fertilizers, both individual and combined strains of *B. amyloliquefaciens* Z2 and *T. harzianum* T22, against cucumber *Fusarium* wilt (*Fusarium oxysporum* f. sp. *cucumerinum*) under this reduced chemical fertilization regime.

## 2. Results

### 2.1. Microbial Inoculants Reduced the Disease Index of Cucumber Under Greenhouse Conditions

**Figure 1:**
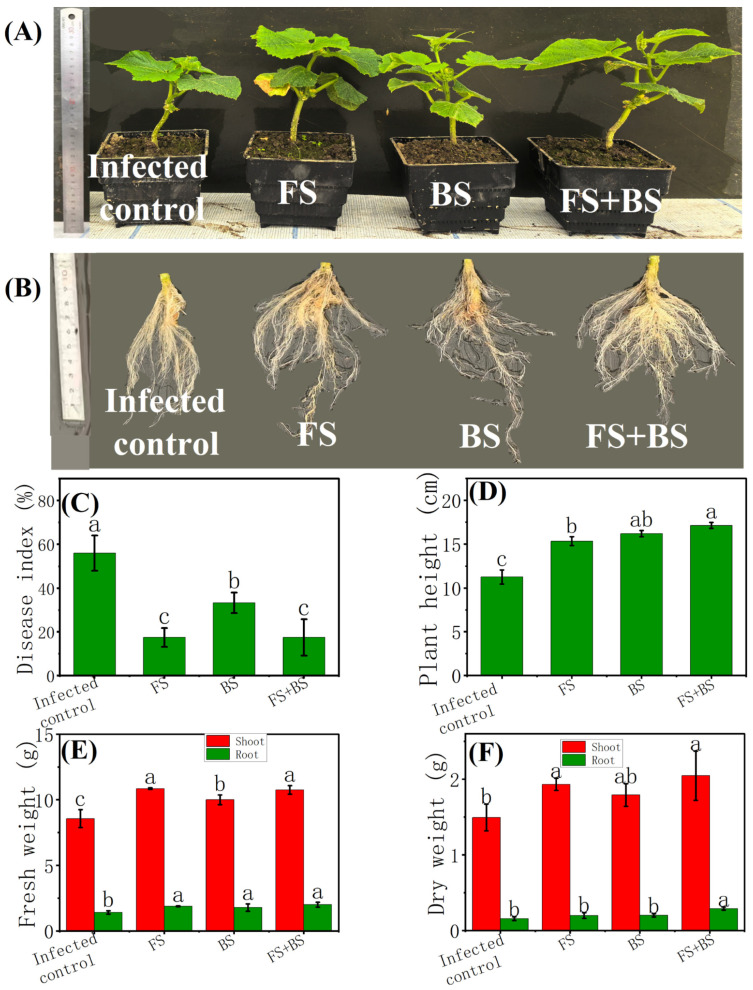
Phenotypic images (**A**), root phenotypic images (**B**), disease index (**C**), plant height (**D**), fresh weight (**E**), and dry weight (**F**) of cucumber in greenhouse experiment. Infected control, no addition of any beneficial microbial agent, inoculated with *F. oxysporum*; FS, root irrigation treatment with fungal spore suspension, inoculated with *F. oxysporum*; BS, root irrigation treatment with bacteria suspension, inoculated with *F. oxysporum*; FS + BS, root irrigation treatment with fungal spore and bacteria solution, inoculated with *F. oxysporum*. The significant difference among different treatments is marked with different letters (*p* < 0.05, Duncan, n = 5).

In the greenhouse experiment, the impacts of *B. amyloliquifaciens*, *T. harzianum*, and their combinations on cucumber disease index and growth promotion were explored and are illustrated in Figure 1. All individual and combined microbial treatments significantly reduced cucumber disease index, and the treatments of fungi solution (FS) and fungi and bacteria solution (FS + BS) exhibited the highest efficacy, resulting in a DI of only 17.50% after 30 days’ transplantation (Figure 1C). In addition, *B. amyloliquifaciens*, *T. harzianum*, and their combinations all significantly increased cucumber seedling plant height by 36.09%, 43.79%, and 52.07% relative to the infected control, respectively (Figure 1D). The treatments of FS, bacteria solution (BS), and FS + BS also notably enhanced cucumber fresh weight by 26.75%, 16.86%, and 25.62% (Figure 1E), and the trend of cucumber fresh root weight followed the order of FS + BS ≈ FS > BS > Infected control (Figure 1E). Moreover, the combined treatment FS + BS also yielded the highest value in cucumber dry weight, with significant increases of 36.94% in cucumber dry shoot weight, and 87.10% in dry root weight compared with the infected control (Figure 1F). Hence, the above results demonstrate that *B. amyloliquifaciens*, and *T. harzianum*, especially their combinations, notably suppress cucumber wilt and increase cucumber growth.

### 2.2. Microbial Bio-Fertilizers Alleviated the Disease Index of Cucumber Under Field Conditions

**Figure 2:**
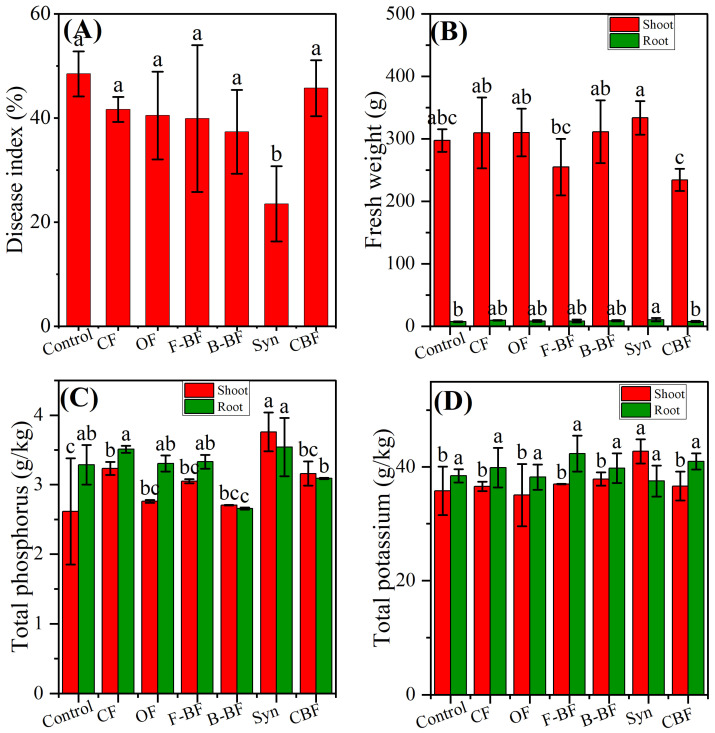
Disease index (**A**), fresh weight (**B**), total phosphorus (**C**), and total potassium (**D**) of cucumber in the field experiment. The field soil was naturally infested with *F. oxysporum*. Control, no fertilizer; CF, mineral fertilizers with conventional rate; OF, chemical N fertilizer reduction by 25% + 7500 kg/hm^2^ organic fertilizer; F-BF, chemical N fertilizer reduction by 25% + 7500 kg/hm^2^ fungi bio-fertilizer; B-BF, chemical N fertilizer reduction by 25% + 7500 kg/hm^2^ bacteria bio-fertilizer; Syn, chemical N fertilizer reduction by 25% + 7500 kg/hm^2^ fungi and bacteria combined bio-fertilizer; CBF, chemical fertilizer reduction by 25% + 7500 kg/hm^2^ commercial bio-fertilizer. The significant difference among different treatments is marked with different letters (*p* < 0.05, Duncan, n = 3).

Under the condition of a constant total nitrogen input (75% chemical, 25% organic/bio-organic), this study builds on previous findings that such a 75:25 ratio improves cucumber yield and quality [24]. This study further investigates the effects of substituting 25% of chemical nitrogen with organic or different bio-fertilizers on cucumber disease suppression, yield, and quality under field conditions in a single growing season from May to September in 2024. The mean DI was decreased in all treatments compared to the control, with DI values of 41.64% for the application of mineral fertilizers (CF), 40.46% for the application→n of organic fertilizer (OF), 39.89% for the application of fungi bio-fertilizer (F-BF), 37.33% for the application of bacteria bio-fertilizer (B-BF), 23.49% for the application of synthetic bio-fertilizer (Syn), and 45.71% for the application of commercial bio-fertilizer (CBF), while the DI of the control was 48.48% (Figure 2A). In addition, the treatments of CF, OF, B-BF, and Syn also increased cucumber shoot weight by 4.08%, 4.27%, 4.73%, and 12.19%, as well as improved fresh root weight from 15.55% to 44.36% compared with the control (Figure 2B). Moreover, there was no significant difference between all treatments in cucumber dry weight (Figure S1). Among these, the treatment of Syn exhibited the best disease control efficacy.

### 2.3. Effect of Microbial Bio-Fertilizers on Cucumber Nutritive Element Content and Soil Chemical Properties

The content of the cucumber three main elements, including total nitrogen (TN), total potassium (TK), and total phosphorus (TP), was measured and is depicted in Figure 2 and Figure S2. The results indicated that the treatment of CF notably increased cucumber shoot and root nitrogen content by 27.65%, and 9.77% relative to the control, respectively, and the similar trends occurred in TP (Figure S2 and Figure 2C). The nitrogen content in other organic fertilizer treatments in cucumber shoot and root was lower than that in CF, except for the treatment of CBF in root (Figure S2). There were no significant differences among the treatments using organic fertilizers and the treatment using mineral fertilizers (CF) in TP and TK in cucumber shoot, except for Syn, and the application of combined bio-fertilizers resulted in a significant increase of 16.29%, and 16.84% in TP and TK relative to CF, respectively (Figure 2C,D). The order of TP content in cucumber root was as follows: Syn ≈ CF > F-BF ≈ OF ≈ Control > CBF > B-BF (Figure 2C), and there were no remarkable differences among all treatments in the concentration of total potassium in cucumber root (Figure 2D).

The impacts of microbial bio-fertilizers on soil properties are listed in Table 1. The results indicated that the applications of fertilizers reduced soil pH from 7.01% to 11.42% compared to the control, and the treatment of Syn exhibited the highest value among the fertilized treatments (Table 1). There were no significant differences in soil OM, (alkaline nitrogen) AN, available phosphorus (AP), available potassium (AK), and cation exchange capacity (CEC) between the control and CF. The OM values of OF, F-BF, B-BF, Syn, and CBF treatments were observably higher compared with those in CF treatment by 4.68%, 5.46%, 4.48%, 6.58%, and 4.68%, respectively (Table 1). There were also no notable differences in soil OM among microbial fertilizers, including OF, F-BF, B-BF, Syn, and CBF (Table 1). The treatments of F-BF, B-BF, and Syn increased soil AP from 1.63% to 12.68% relative to CF, respectively, and only the application of combined bio-fertilizer (Syn) significantly increased soil AP by 12.68% compared with CF (Table 1). The treatments of OF, F-BF, B-BF, Syn, and CBF remarkably enhanced soil AK by 31.43%, 38.21%, 21.42%, 15.39%, and 40.15% compared with CF (Table 1). In addition, the applications of organic fertilizers had no notable positive effects on soil CEC relative to CF (Table 1).

### 2.4. Effect of Microbial Bio-Fertilizers on Cucumber Yield and Nutritional Quality

**Figure 3:**
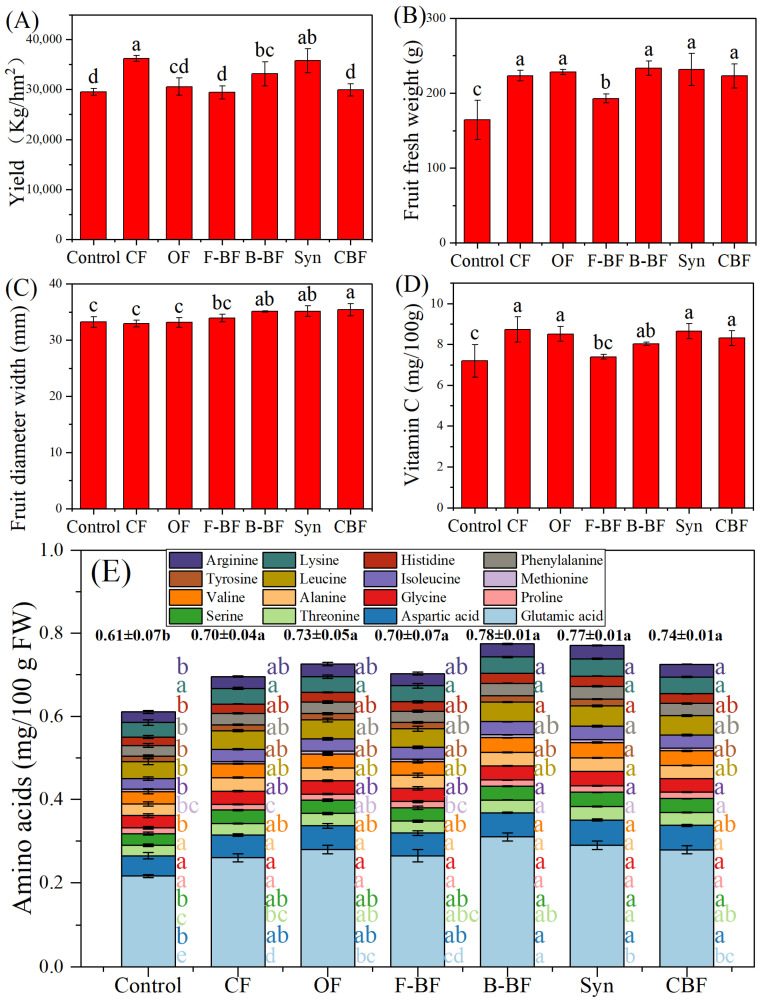
Yield (**A**), fruit fresh weight (**B**), fruit diameter width (**C**), vitamin C (**D**), and amino acids (**E**) of cucumber in the field experiment. The field soil was naturally infested with *F. oxysporum*. Control, no fertilizer; CF, mineral fertilizers with conventional rate; OF, chemical N fertilizer reduction by 25% + 7500 kg/hm^2^ organic fertilizer; F-BF, chemical N fertilizer reduction by 25% + 7500 kg/hm^2^ fungi bio-fertilizer; B-BF, chemical N fertilizer reduction by 25% + 7500 kg/hm^2^ bacteria bio-fertilizer; Syn, chemical N fertilizer reduction by 25% + 7500 kg/hm^2^ fungi and bacteria combined bio-fertilizer; CBF, chemical fertilizer reduction by 25% + 7500 kg/hm^2^ commercial bio-fertilizer. The significant difference among different treatments is marked with different letters (*p* < 0.05, Duncan, n = 3).

Regarding yield, cucumber yield for the treatments of CF, OF, F-BF, B-BF, Syn, and CBF was recorded as 36,225 kg/ha^−1^, 30,566 kg/ha^−1^, 29,421 kg/ha^−1^, 33,134 kg/ha^−1^, 29,913 kg/ha^−1^, compared with only 29,840 kg/ha^−1^ in the control (Figure 3A). The application of mineral fertilizers alone significantly improved cucumber fruit fresh weight, fruit length, vitamin C, and total amino acids by 36.07%, 13.11%, 21.28%, and 14.75% relative to the control (Figure 3B,D,E and Figure S3). There were no notable differences among CF, OF, Syn, and CBF in fruit fresh weight, fruit length, vitamin C, and total amino acids (Figure 3B,D,E and Figure S3). The treatments of B-BF, Syn, and CBF observably enhanced cucumber fruit diameter width by 6.56%, 6.65%, and 7.40% compared with CF (Figure 3C). In addition, OF, B-BF, Syn, and CBF also increased total amino acids content by 4.29%, 11.43%, 10.00%, and 5.71% relative to CF (Figure 3E, Table S1). More specially, 16 amino acids were identified in cucumber fruits, and CF improved 15 of 16 identified amino acids to some extent compared with the control, with the exception of methionine (Figure 3E, Table S1). Moreover, the treatments of B-BF and Syn also increased the majority of amino acid content from 1.53% to 31.82% relative to CF, except for the content of alanine (Figure 3E, Table S1).

### 2.5. Effect of Microbial Bio-Fertilizers on Cucumber Rhizosphere Soil Bacterial Community

**Figure 4:**
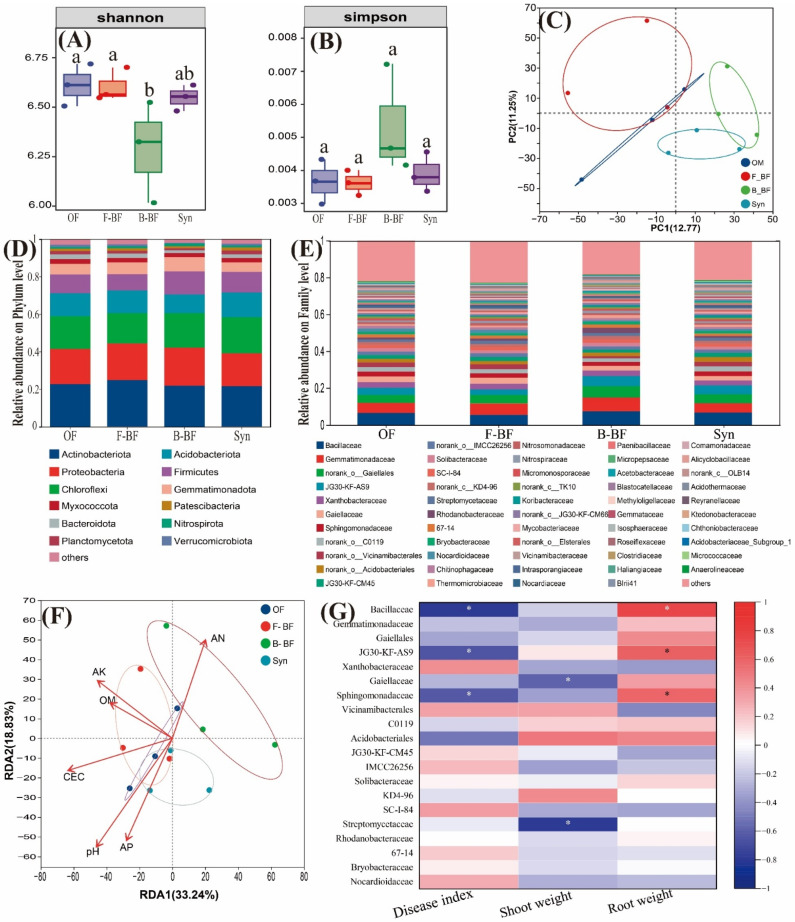
Alpha diversity (**A**,**B**), PcoA (**C**), the relative abundance of a certain phylum level (**D**) and family level (**E**), RDA (**F**) of soil bacterial communities in cucumber rhizosphere soil. The correlation analysis of the relative abundance of cucumber rhizosphere soil bacterial communities at the family level with cucumber *Fusarium* wilt index, shoot weight, and root weight (**G**). The field soil was naturally infested with *F. oxysporum*. OF, chemical N fertilizer reduction by 25% + 7500 kg/hm^2^ organic fertilizer; F-BF, chemical N fertilizer reduction by 25% + 7500 kg/hm^2^ fungi bio-fertilizer; B-BF, chemical N fertilizer reduction by 25% + 7500 kg/hm^2^ bacteria bio-fertilizer; Syn, chemical N fertilizer reduction by 25% + 7500 kg/hm^2^ fungi and bacteria combined bio-fertilizer. The significant difference among different treatments is marked with different letters (*p* < 0.05, Duncan, n = 3). Arrow length indicates the relative strength (explanatory power) of the environmental factor in RDA. Significant correlations are indicated by asterisks (* *p* < 0.05) in Figure 4G.

Given the excellent effects of organic fertilizers in controlling cucumber wilt disease, all treatments with organic fertilizers added were selected to explore their impact on the soil microbial community. Analysis of the bacterial community’s alpha diversity in the cucumber rhizosphere at harvest showed that the index of Shannon followed the order of B-BF < Syn < F-BF ≈ OF (Figure 4A). Conversely, the Simpson index of B-BF was higher than that in the other treatments, but not notably (Figure 4B). For the bacterial community, the first two principal coordinates in the principal coordinates analysis (PCoA) explained 12.77% and 11.25% of the variance, respectively. The results of PcoA showed that there was an overlap between B-BF and Syn, but the treatments of B-BF and Syn were completely separated from the other two organic fertilizer treatments (Figure 4C).

The composition of bacterial communities at the phylum level was illustrated in Figure 4D, and *Actinobacteriota*, *Proteobacteria*, *Chloroflexi*, *Acidobacteriota*, and *Firmicutes* were the dominant bacteria in all treatments (Figure 4D and Figure S4). The order of the relative abundance of *Actinobacteriota* was as follows: F-BF > OF ≈ B-BF > Syn, and Syn exhibited the highest values in the relative abundance of *Firmicutes* (Figure S4). There were no significant differences among organic fertilizers treatments in the other top three bacterial phyla. The family-level bacterial community composition was exhibited in Figure 4E, and B-BF exhibited the highest values in the top four families (Figure 4E and Figure S5).

The relationship between soil chemical properties and microbial community composition was conducted based on the redundancy analysis (RDA), and the first two axes in RDA together explained 52.07% of the total variance in soil bacterial community (axis 1: 33.24%, and axis 2: 18.83%) (Figure 4F). RDA showed that the order of the correlation between soil properties and soil bacterial community was as follows: pH > CEC > AP > AN > AK > OM (Figure 4F). The correlation between the relative abundance of soil bacterial community at the family level and the cucumber wilt incidence was performed based on Spearman’s correlation analysis and is illustrated in Figure 4G. Cucumber wilt disease index was observably negatively associated with the relative abundance of *Bacillaceae*, JG30-KF-AS9, and *Sphingomonadaceae* (*p* < 0.05). On the contrary, cucumber root weight was significantly positively correlated with the relative abundance of the above three families (Figure 4G). In addition, the relative abundance of *Gaiellales*, and *Streptomycetaceae* was also observably negatively correlated with cucumber shoot weight (*p* < 0.05).

### 2.6. Effect of Microbial Bio-Fertilizers on Cucumber Rhizosphere Soil Fungal Community

**Figure 5:**
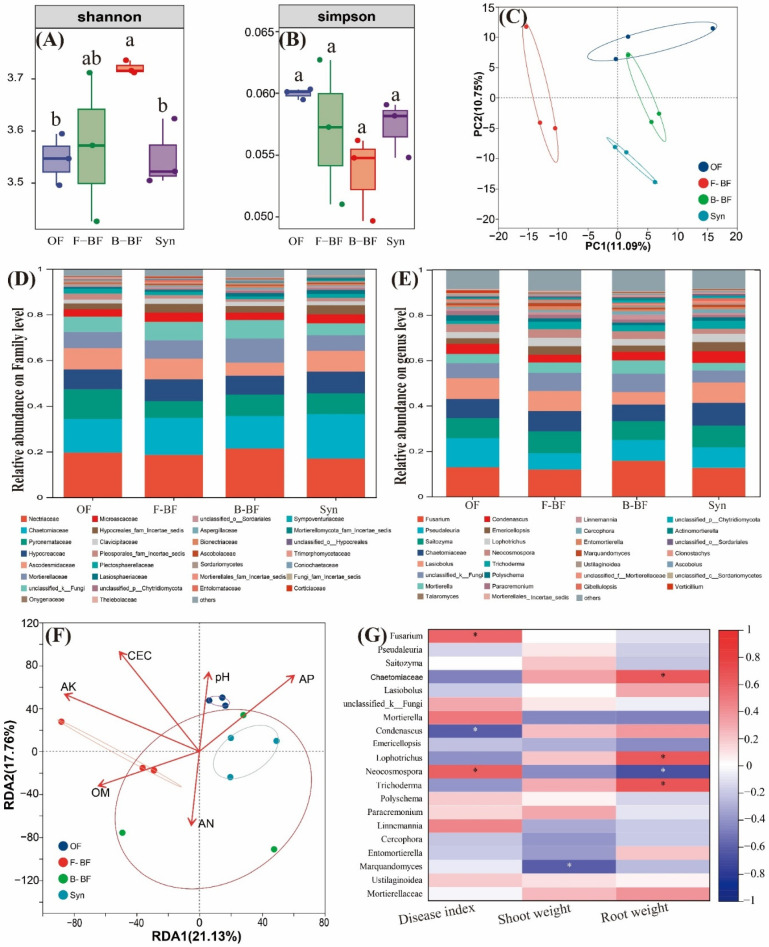
Alpha diversity (**A**,**B**), PcoA (**C**), the relative abundance of a certain family level (**D**) and genus level (**E**), and RDA (**F**) of soil fungal communities in cucumber rhizosphere soil. The correlation analysis of the relative abundance of cucumber rhizosphere soil fungal communities at the genus level with cucumber *Fusarium* wilt index, shoot weight, and root weight (**G**). The field soil was naturally infested with *F. oxysporum*. OF, chemical N fertilizer reduction by 25% + 7500 kg/hm^2^ organic fertilizer; F-BF, chemical N fertilizer reduction by 25% + 7500 kg/hm^2^ fungi bio-fertilizer; B-BF, chemical N fertilizer reduction by 25% + 7500 kg/hm^2^ bacteria bio-fertilizer; Syn, chemical N fertilizer reduction by 25% + 7500 kg/hm^2^ fungi and bacteria combined bio-fertilizer. The significant difference among different treatments is marked with different letters (*p* < 0.05, Duncan, n = 3). Arrow length indicates the relative strength (explanatory power) of the environmental factor in RDA. Significant correlations are indicated by asterisks (* *p* < 0.05) in Figure 5G.

The results of the fungal community alpha diversity analysis are displayed in Figure 5A,B, and the trend of the Shannon index followed the order of B-BF > F-BF ≈ OF ≈ Syn. No significant differences were observed among all treatments in the Simpson index. PcoA was also selected to display the fungal community structure, and the first two axes in PcoA analysis explained 21.84% of the total variance (axis 1: 11.09%, and axis 2: 10.75%). More specifically, the treatments with *T. harzianum* added (including F-BF and Syn) were notably separated from the other two treatments (Figure 5C).

The family-level composition of fungal communities is presented in Figure 5D, and *Nectriaceae*, *Chaetomiaceae*, *Pyronemataceae*, *Hypocreaceae*, and *Ascodesmidaceae* are the dominant fungi (Figure S6). The trend of the relative abundance of *Nectriaceae* followed the order of B-BF > OF ≈ F-BF > Syn, where Syn exhibited the highest values in the relative abundance of *Chaetomiaceae* (Figure S6). OF significantly increased the relative abundance of *Pyronemataceae* compared to the other three treatments, and there were no significant differences in the relative abundance of *Hypocreaceae* among all treatments (Figure S6). Moreover, the trend of *Ascodesmidaceae* followed the order of OF ≈ Syn ≈ F-BF > B-BF. Subsequently, the changes in the genus level in the fungal composition were also analyzed (Figure 5E), and the relative abundance of *Fusarium* in B-BF treatment and that of *Pseudaleuria* in OF treatment were observably higher than those in other treatments (Figure S7). In addition, B-BF exhibited the lowest values in the relative abundance of *Saitozyma*, *Chaetomiaceae*, and *Lasiobolus*, whereas the application of combined bio-fertilizers exhibited the best performance in *Chaetomiaceae* (Figure S7). Moreover, the exogenously added *T. harzianum* was also detected at the genus level, and the relevant results indicated that the trend of the relative abundance of *Trichoderma* followed the order of Syn > F-BF > B-BF > OF (Figure S7).

RDA was performed to examine the correlation of soil chemical properties and fungal community composition, and the first two axes together explained 38.89% of the total variance (axis 1: 21.13%, and axis 2: 17.76%) (Figure 5F). AK and CEC of the soil properties exhibited the greatest impact on the fungal community (*p* < 0.05), followed by AP, OM, pH, and AN (Figure 5F). Spearman analysis was conducted to show the relationship of the top twenty genera and cucumber DI, as well as cucumber fresh biomass, and the results indicated that cucumber DI was notably positively associated with the relative abundance of *Fusarium* and *Neocosmospora*, but negatively correlated with that of *Condenascus* (Figure 5G). Cucumber fresh shoot weight was only significantly negatively correlated with the relative abundance of *Marquandomyces*. Moreover, the relative abundance of soil fungal *Chaetomiaceae*, *Lophotrichus*, and *Trichoderma* was remarkably positively associated with cucumber root weight (Figure 5G). The relative abundance of *Neocosmospora* showed a trend opposite to the above three genera in cucumber root (*p* < 0.05) (Figure 5G).

## 3. Discussion

### 3.1. Microbial Bio-Fertilizers Improved Cucumber Growth and Alleviated Fusarium Wilt

The severe threat of plant diseases is one of the challenges in vegetable cultivation [8]. Previous studies have reported that cucumber *Fusarium* wilt, which is investigated in this study, can cause yield losses of up to 80% [8]. On the other hand, a recent survey study has found that the application rate of chemical fertilizers for vegetables in China is 3.3 times as high as that of crops in China [4]. While chemical fertilizers can boost yields, their excessive use not only wastes resources but also causes environmental pollution, such as water eutrophication [26]. Furthermore, existing research has indicated that heavy reliance on chemical fertilizers is often ineffective in reducing cucumber disease index [27]. Hence, the reduction of chemical fertilizer usage while enhancing its effectiveness is also a significant challenge faced by vegetable cultivation. Currently, numerous studies have demonstrated that organic fertilizers can not only partially replace chemical fertilizers in supplying nutrients to crops but also play a positive role in helping plants combat stresses like crop diseases [18,28,29]. These findings have been corroborated by our laboratory’s previous research, which showed that substituting 25% nitrogen of chemical fertilizers with organic fertilizer improved cucumber yield and quality [24]. Based on this foundation, our current experiment introduced two beneficial microorganisms to create bio-organic fertilizers, with subsequent investigation of the effects of applying these bio-organic fertilizers, both individually and in combination, on cucumber disease suppression, yield, nutritional quality, and soil microbial community.

Both *B. amyloliquefaciens* and *T. harzianum* were reported to be effective in promoting crop growth and suppressing cucumber wilt, supporting previous studies on their ability to act as growth stimulators and biocontrol agents [30,31,32]. In this paper, the results of greenhouse experiments illustrated that individual and combined microbial inoculants alleviated cucumber wilt, and Syn exhibited the best performance according to cucumber DI, cucumber height, fresh weight, and dry weight (Figure 1). In field experiments, the application of chemical fertilizers (CF) decreased DI by 14.11%, but not significantly compared to the control (Figure 2). Subsequently, the application of substituting 25% of chemical nitrogen with organic or different bio-organic fertilizers containing the aforementioned microbial inoculants not only increased cucumber growth but also alleviated cucumber wilt severity, resulting in the enhancements of cucumber yield and quality (Figure 2 and Figure 3), consistent with the previous literature [21,33]. In addition, our study also confirmed that the synthetic bio-organic fertilizer remained effective in promoting cucumber growth, cucumber yield, and resisting stress even with a substitution for 25% of chemical nitrogen fertilizer. Moreover, both the disease index (DI) and cucumber weight in the Syn treatment were also found to be higher than those in CBF (Figure 2A,B). This could be due to more intense competition among the similar *Bacillus* strains within CBF, compared to the bacterial–fungal interactions in Syn. However, the field trial in this study only lasted for one year, so there are still certain limitations in comprehensively understanding the impacts of using bio-fertilizer as a substitute for 25% of nitrogen fertilizer on the wilt disease of cucumbers.

### 3.2. Microbial Bio-Fertilizers Optimized Cucumber Nutritive Element Content and Soil Chemical Properties

The beneficial microorganisms can not only promote crop growth and yield but also play a positive role in altering crop and soil nutrient cycling [34]. Our findings reveal that mineral fertilizers at the normal level significantly enhanced cucumber nitrogen content, and there were no notable differences in cucumber root nitrogen content between the combined treatment and mineral fertilizers treatment (Figure S2). Hence, the above results indicate that when 25% of nitrogen fertilizer was replaced with bio-fertilizer, the compound bio-fertilizer could also provide sufficient nitrogen for cucumber growth in a timely manner, compensating for the adverse effects of insufficient chemical nitrogen fertilizer application on cucumber development [28]. *Bacillus* was usually reported to be able to dissolve P and K-minerals, which finally resulted in the improvement of P and K content in crops [35,36]. Biocontrol agent *Trichoderma* could also solubilize rock phosphate (mostly calcium phosphate), with subsequent improvement of P content in crop [37,38]. Although the application of the above *Bacillus* or *Trichoderma* bio-fertilizer alone had little effect on P and K content in cucumbers, this study observed that the combined treatment significantly increased the levels of P and K in the cucumber shoot, compared to conventional mineral fertilization (CF) (Figure 2C,D).

The impacts of microbial bio-fertilizers on soil chemical properties were subsequently analyzed, and the application of combined bio-organic fertilizers notably improved soil AP and AK content relative to CF, which was consistent with the content of P and K in cucumber (Table 1). The optimization of nutrient availability is essential for robust plant growth, and microbial inoculants contribute significantly to enhancing rhizosphere nutrient cycling as they secret compounds that solubilize minerals into bioavailable forms [39]. Hence, the above results indicated that the partial substitution (25% N) of chemical fertilizers with bio-fertilizers enhanced the availability of soil AP and AK through microbial activity, thereby improving cucumber P and K uptake and, ultimately, promoting plant growth and disease resistance. Moreover, our findings also illustrated that the application of chemical fertilizers could notably reduce soil pH, consistent with previous studies [40]. Furthermore, although this experiment was conducted for only one year, our preliminary findings demonstrated that the application of organic fertilizer significantly enhanced soil OM, which was consistent with previous research [41]. Hence, the addition of combined bio-fertilizers can not only optimize soil fertility and nutrient release but also have positive effects on soil OM, resulting in the enhancements of crop nutritive level and yield quality.

### 3.3. Microbial Bio-Fertilizers Reshaped Soil Microbial Community Composition

On the other hand, rhizosphere microbial communities act as a major driver of enhancing crop growth and preventing crop diseases [42]. The α-diversity measurements in this paper exhibited a similar trend among all treatments in the bacterial and fungal microbial community (Figure 4A,B and Figure 5A,B). Specifically, the individual application of *Bacillus* and *Trichoderma* bio-organic fertilizers resulted in the lowest Shannon indices in both bacterial and fungal communities, whereas the Simpson indices were the largest but not statistically significant compared with OF. No significant differences were observed in the aforementioned two indices between Syn and OF treatments. These findings indicate that the single application of either *Bacillus* or *Trichoderma* bio-fertilizer had a greater impact on the evenness of bacterial and fungal communities in cucumber rhizosphere soil compared to the combined treatment. The underlying reason may be attributed to the competitive interaction between *Bacillus* and *Trichoderma* in the composite treatment, which potentially influenced their respective performances in the synthetic treatment [21].

Although there were no significant differences between Syn and OF treatments in the relative abundance of *Fusarium*, DI in the treatment of OF was notably higher than that in Syn (Figure S7). The stronger disease suppressive effect of synthetic treatment could be attributed to the enrichment of beneficial microorganisms resulting from the application of the composite microbial inoculant, such as JG30-KF-AS9 and *Sphingomonadaceae*, and beneficial fungal genera, such as *Chaetomiaceae*, *Condenascus*. Interestingly, the relative abundance of JG30-KF-AS9, *Sphingomonadaceae*, and *Bacillaceae* (the family of the inoculated *Bacillus*) was negatively correlated with cucumber DI and positively associated with cucumber root weight (Figure 4G). Moreover, the relative abundance of *Condenascus* was also negatively associated with cucumber DI, whereas *Chaetomiaceae* was positively correlated with cucumber root weight (Figure 5G). The above microorganisms could play positive roles in nutrition cycling and organic matter decomposition, resulting in crop growth and health. For example, JG30-KF-AS9 was reported to contribute significantly to the variation in bacterial communities between fertilizer-reducing and control treatments. Furthermore, its abundance was also positively correlated with tobacco growth [43]. The beneficial *Sphingomonadaceae* family, which is recruited by supernodulating soybean plants through the secretion of oleic acid and cis-4-hydroxy-D-proline, significantly enhanced rapeseed growth when applied exogenously, either alone or in combination with rhizobia [44]. Moreover, as a beneficial genus within the Ascomycota, *Chaetomiaceae* was identified as a producer of both antifungal compounds and cellulose, suggesting a role in suppressing soil-borne diseases [45]. *Condenascus* was also reported to be positively correlated with tobacco growth [46]. In addition, although the relative abundance of beneficial *Trichodema* added in the field experiment was not negatively correlated with cucumber DI, it was notably positively associated with cucumber root weight (*p* ≤ 0.05). The growth-promoting effects of *Trichoderma harzianum* have been reconfirmed in this study; however, its disease suppression may not have been fully realized, potentially due to the short duration of application. In support of this, Huang et al. (2011) reported that extending the application of *Trichoderma harzianum* SQR-T37 enhanced its disease control efficacy from 45% in the first growing season to 82% in the second season, underscoring the importance of application duration for the effectiveness of microbial bio-fertilizers in disease management [47]. Hence, these findings reveal that the rhizosphere microbiota, shaped by microbial inoculants, plays a crucial role in modulating crop growth and pathogen suppression.

## 4. Materials and Methods

### 4.1. Strains and Growth Conditions

The bacterial and fungal strains in this study were supplied by Chengdu Siyou Biotechnology Co., Ltd. (Chengdu, China). *B. amyloliquefaciens* Z2 was grown at 37 °C in a lysogeny broth medium (tryptone ten g/L, NaCl five g/L, and yeast extract five g/L). Strains in the exponential phase were washed three times with sterilized water by centrifugation (6000 rpm, 25 °C, 10 min), with subsequent dilution to a concentration of 1 × 10^8^ cfu/mL. *T. harzianum* T22 was cultured on the potato dextrose agar medium (PDA) at 30 °C for seven days. After 7 days’ incubation, the spores were then harvested by washing the plates with sterile deionized water. The suspension was filtered through a double layer of sterile gauze to remove mycelial fragments, and the spore concentration was determined using a hemocytometer under a microscope. (Leica DM1000 LED, Leica Microsystems, Wetzlar, Germany). The cucumber (*Cucumis sativus* L.) cultivar “Chuancui No.3”, a wilt-susceptible genotype, was used in this study. The soil-borne fungal pathogen *F. oxysporum* f. sp. *cucumerinum* NJAU-2 (FOC NJAU-2), which causes cucumber wilt, was provided by Jiangsu Provincial Key Lab (Nanjing, China) for Organic Solid Waste Utilization, Nanjing Agricultural University. Fungal colonies were inoculated onto PDA plates and incubated at 37 °C for 7 days. Spores were then harvested by washing with boiled deionized water, filtered through sterile gauze to remove mycelial fragments, and counted under a microscope.

### 4.2. Pot Experiments

The fresh infected soil in this experiment was obtained from a vegetable field (Youfu farm) in Chengdu, China, and the plots were cultivated with cucumber for five consecutive seasons, exhibiting notable signs of cucumber wilt disease. The soil was subsequently air-dried, ground, and passed through a 20-mesh sieve before use. A total of 600 g dry soil was distributed into each pot (10 × 10 cm), and the pathogen spore suspension was mixed with soils to a pathogen density of 1 × 10^6^ spores/g dry soil [21]. All treatments were inoculated with *F. oxysporum*. In this study, pot experiments were performed to evaluate the effect of cooperating strains on the efficacy of cucumber wilt suppression, which contained four treatments: (1) infected control, no addition of any beneficial microbial agent, inoculated with *F. oxysporum*; (2) FS, root irrigation treatment with fungal spore suspension, inoculated with *F. oxysporum*; (3) BS, root irrigation treatment with bacteria suspension, inoculated with *F. oxysporum*; (4) FS + BS, root irrigation treatment with fungal spore and bacteria solution, inoculated with *F. oxysporum*. The suspension of *B. amyloliquefaciens* Z2 and *T. harzianum* T22 was irrigated to a final ratio of 1 × 10^6^ cfu/g dry soil and 1 × 10^6^ spores/g dry soil in each treatment, respectively [21].

Prior to harvest, the disease index in cucumbers was recorded based on symptom severity. The disease severity was assessed on a 1–5 scale, where 1 = no symptoms, 2 = slight stunning, 3 = stunning or partially wilting, 4 = completely wilting, and 5 = crop death. The disease index was calculated according to the following equation [48]:

$$\mathrm{disease index}=\frac{\sum \left(Si\times Ni\right)}{S\times N}$$

where *Si* represents the symptoms rating, *Ni* is the count of cucumbers exhibiting rating *Si*, *S* is the highest symptoms rating (5), and *N* is the total cucumber count within the treatment.

After one month of growth, cucumber height and the fresh weight of the cucumber were recorded. In addition, the images of cucumber root morphology were also obtained. The plants were dried for two days at 70 °C, and the dry weight of the cucumber was measured.

### 4.3. Field Experiments

The field trial was located at Youfu farm in Pengzhou County, Chengdu City, Sichuan Province, and the plots were cultivated with cucumber for five consecutive seasons and exhibited notable signs of cucumber wilt disease. Additionally, the soil used in the pot experiment in this paper belongs to the same plot in the field experiment. A field trial was performed in a subtropical humid monsoon climate zone, with an annual average precipitation of 885.40 mm and an average annual temperature of 16.4 °C. The soil had an OM content of 35.60 g/kg, a pH of 5.60, and contained 189.00 mg/kg of AN, 303.00 mg/kg of AK, and 260.90 mg/kg of AP. The tested organic fertilizer was provided by Sichuan Siyou Biotechnology Co., Ltd. (Chengdu, China), and it was made from pig manure, with N content of 15.50 g/kg, P_2_O_5_ content of 31.00 g/kg, K_2_O content of 25.00 g/kg, OM content of 405.00 g/kg, and moisture content of 21.42%. A field validation trial was conducted in a single growing season in 2024 from May to September. Throughout the trial period, no chemical fungicides or pesticides were applied for disease management. Our previous study found that the application of 75% chemical fertilizer nitrogen combined with 25% organic fertilizer nitrogen significantly enhanced cucumber yield and quality [24]. Therefore, based on previous findings, this study further investigated the effects of substituting 25% of chemical nitrogen with organic or different bio-organic fertilizers on cucumber disease suppression, yield, and quality.

Hence, seven treatments were applied: (1) control, no fertilizer; (2) CF, NPK dosage: 150.00 kg N/hm^2^: 105.00 kg P/hm^2^: 300.00 kg K/hm^2^; (3) OF, chemical N fertilizer reduction by 25% + 7500 kg/hm^2^ organic fertilizer; (4) F-BF, chemical N fertilizer reduction by 25% + 7500 kg/hm^2^ fungi bio-fertilizer; (5) B-BF, chemical N fertilizer reduction by 25% + 7500 kg/hm^2^ bacteria bio-fertilizer; (6) Syn, chemical N fertilizer reduction by 25% + 7500 kg/hm^2^ fungi and bacteria bio-fertilizer; (7) CBF chemical fertilizer reduction by 25% + 7500 kg/hm^2^ commercial bio-fertilizer (Table S2). CBF, which contains *Bacillus subtilis*, *Bacillus megaterium*, and *Bacillus licheniformis*, was supplied by Sichuan Meishan Yiji Agricultural Science and Technology Co., Ltd. (Meishan, China). The total nitrogen input was kept constant, with 75% supplied by chemical fertilizer and 25% by organic or bio-organic fertilizer. Each treatment was replicated three times, resulting in a total of 21 experimental plots. These plots, each covering an area of 20 m^2^ and planted with 50 cucumber seedlings, were arranged randomly. The total application rates of N, P, and K were 150 kg/hm^2^, 105 kg/hm^2^, and 300 kg/hm^2^, respectively (including contributions from organic fertilizer, which was applied once as a basal dose; any deficit was supplemented with chemical fertilizers). Organic fertilizer and calcium superphosphate were applied once as basal fertilizer, while nitrogen and potassium fertilizers were split equally between basal and topdressing applications at a 1:1 ratio. Cucumbers were planted on 1.5-m-wide raised beds, with two rows per bed and a plant spacing of 0.40 m. After 80 days of transplanting the seedlings, DI in cucumbers was recorded in the field experiment according to Section 4.2, followed by the harvest of the cucumber plants and fruits.

### 4.4. Rhizosphere Soil Sampling and DNA Extraction

After 80 days of transplanting the seedlings, rhizosphere soil samples were collected, as described by Tao et al. (2020) [29]. Specifically, three cucumber root systems were collected from each plot. The soil adhering to the root surfaces was shaken off, and the roots were cut into approximately 4 cm long segments. Subsequently, soil tightly adhering to roots was recovered via sterile saline rinse, and the resulting suspension was subsequently centrifuged (10 min, 10,000× *g*), which was defined as rhizosphere soil. Finally, twenty-one (seven treatments with three replicates each) rhizosphere samples underwent storage at −80 °C prior to DNA extraction. Total soil genomic DNA was extracted from 0.5 g rhizosphere soil samples using the E.Z.N.A.^®^ soil DNA Kit (Omega Bio-tek, Norcross, GA, USA) following the manufacturer’s protocol. DNA concentration and purity were measured using a NanoDrop 2000 spectrophotometer (Thermo Scientific, NanoDrop 2000 software, Waltham, MA, USA). Bacterial and fungal communities were analyzed by paired-end amplicon sequencing of the 16S rRNA gene and the ITS region, respectively, using an Illumina MiSeq PE 250 platform at Meiji Biotechnology Co., Ltd. (Shanghai, China). The bacterial 16S rDNA V3–V4 region was PCR amplified using 338F (5′-ACTCCTACGGGAGGCAGCAG-3′) and 806R (5′-GGACTACHVGGGTWTCTAAT-3′), and the fungal ITS1 region was PCR amplified using ITS1F (5′-CTTGGTCATTTAGAGGAAGTAA-3′) and ITS1R (5′-GCTGCGTTCTTCATCGATGC-3′).

### 4.5. Bulk Soil Sampling and Analysis

Bulk soil samples were obtained according to Tao et al. (2020) [29]. Specifically, bulk soil samples were obtained by removing cucumber plants and then collecting soil cores to a depth of 10 cm after transplantation, with subsequent filtration through a 2 mm mesh. The soil properties were determined according to the following standardized methods: pH (NY/T 1121.2-2006) [49], organic matter (NY/T 1121.6-2006) [50], alkali-hydrolyzable nitrogen (DB51/T 1875-2014) [51], available phosphorus (NY/T 1121.7-2014) [52], available potassium (NY/T 889-2004) [53], and CEC (NY/T 295-1995) [54].

### 4.6. Cucumber Plant Elemental Analysis and Nutritional Analysis

The impacts of different bio-fertilizers on the cucumber plant element levels and nutritional quality were further explored. After 80 days of transplanting the seedlings, cucumber plants and fruits were collected, and fresh plant weight, fresh fruit weight, fruit length, and fruit diameter were measured. The plants were dried for two days at 70 °C, and TN, TK, and TP of cucumber plants were measured according to the following standardized methods: TN (NY/T 2419-2013) [55], TK (NY/T 2420-2013) [56], and TP (NY/T 2421-2013) [57]. Moreover, the content of fresh cucumber fruit vitamin C (Vc) and amino acids (AAs) was determined by the standardized methods: Vc (GB 5009.124-2016) [58], and AAs (GB 5009.86-2016) [59].

### 4.7. Statistical Analysis

Both the greenhouse and field experiments were conducted using a randomized block design, with all measurements performed in at least triplicate. Data are expressed as the mean ± standard deviation. All statistical analyses were performed with R (version 4.3.1). The significant differences were assessed by A one-way analysis of variance (ANOVA) with Duncan test (*p* < 0.05).

## 5. Conclusions

In conclusion, the combined *Bacillus* and *Trichoderma* inoculant was the most effective treatment for suppressing cucumber wilt and enhancing growth. It performed consistently across greenhouse and field conditions, and when integrated with 25% reduced chemical fertilizer, it maintained high disease suppression and increased yield. This mixed bio-fertilizer enriched specific beneficial microbes (e.g., JG30-KF-AS9, *Sphingomonadaceae*) more effectively than single strains, revealing a microbiome-mediated mechanism of pathogen suppression. We conclude that reducing chemical fertilizers while applying mixed bio-fertilizers optimizes soil microbial communities to support plant health, providing a sustainable basis for microbe-driven agricultural practices.

## Figures and Tables

**Table 1:** Chemical properties of soil.

Treatment	pH	Organic Matter (OM)	Alkali-Hydrolyzable Nitrogen (AN)	Available Phosphorus (AP)	Available Potassium (AK)	Cation Exchange Capacity (CEC)
Control	6.13 ± 0.06 a	36.20 ± 0.33 b	190.50 ± 2.50 c	267.10 ± 1.40 c	288.67 ± 3.86 d	11.04 ± 0.17 ab
CF	5.60 ± 0.01 d	35.70 ± 0.14 b	198.00 ± 9.00 bc	266.40 ± 5.50 c	309.67 ± 22.54 d	11.13 ± 0.25 ab
OF	5.67 ± 0.06 bc	37.37 ± 0.58 a	217.00 ± 8.04 a	289.90 ± 7.10 b	407.00 ± 12.00 ab	11.27 ± 0.18 a
F-BF	5.63 ± 0.06 cd	37.65 ± 0.55 a	202.00 ± 6.38 abc	270.73 ± 0.47 c	428.00 ± 36.00 a	11.33 ± 0.03 a
B-BF	5.40 ± 0.01 e	37.30 ± 1.21 a	214.00 ± 21.00 ab	274.37 ± 5.50 c	376.00 ± 14.35 bc	10.81 ± 0.01 bc
Syn	5.70 ± 0.01 c	38.05 ± 0.05 a	206.50 ± 9.50 abc	300.17 ± 9.35 a	357.33 ± 3.86 c	11.01 ± 0.30 ab
CBF	5.43 ± 0.06 e	37.37 ± 0.45 a	212.33 ± 9.74 ab	252.15 ± 8.75 d	434.00 ± 63.00 a	10.67 ± 0.22 c

Note: The significant difference among different treatments is marked with different letters (*p* < 0.05, Duncan, n = 3).

## Data Availability

Data from the experiments are available from the corresponding author upon request.

## Supplementary Materials

The following supporting information can be downloaded at: https://www.mdpi.com/article/10.3390/plants15050782/s1, Figure S1: Dry weight of cucumber in the field experiment; Figure S2: Total nitrogen of cucumber in the field experiment; Figure S3: Fruit length of cucumber in the field experiment; Figure S4: The relative abundance of certain bacterial phylum in the cucumber rhizosphere; Figure S5: The relative abundance of certain bacterial family in the cucumber rhizosphere; Figure S6: The relative abundance of certain fungal family in the cucumber rhizosphere; Figure S7: The relative abundance of certain fungal genus in the cucumber rhizosphere; Table S1: Content of amino acids of cucumber in the field experiment; Table S2: Treatments of cucumber in the field experiment.

## Abbreviations

The following abbreviations are used in this manuscript:

OM	Organic material
AN	Alkaline nitrogen
AP	Available phosphorus
TN	Total nitrogen
TK	Total potassium
TP	Total phosphorus
BCAs	Biocontrol agents
CEC	Cation exchange capacity
RDA	Redundancy analysis
PcoA	Principal coordinates analysis
DI	Disease index
