# Supplementary material for: Combining Bacillus and Trichoderma in Bio-Organic Fertilizers with Reduced Chemical Fertilizer: An Effective Strategy Against Cucumber Fusarium Wilt
Source: Plants (Basel). 2026 Mar 4;15(5):782. doi: 10.3390/plants15050782 (PMC12987329; doi:10.3390/plants15050782)
Supplement: Supplementary file 1 [file plants-15-00782-s001.zip › plants-4150847-supplementary.pdf]

Supplementary Materials

Combining *Bacillus* and *Trichoderma* in Bio-organic Fertilizers with Reduced Chemical Fertilizer: An Effective Strategy against Cucumber *Fusarium* Wilt

Xing Luo [1,2], Jiawei Ouyang [3], Jing Li [4], Hua Yu [1,2], Song Guo [1], Xiangzhong Zeng [1], Zijun Zhou [1], Yuxian Shangguan [1], Mingjiang He [1], Yiting Ouyang [1], Kun Chen [1] and Yusheng Qin [1,*]

[1] Institute of Agricultural Resources and Environment, Sichuan Academy of Agricultural Sciences, Chengdu 610066, China; luoxingjn@scsaas.cn (X.L.); yuhua135@scsaas.cn (H.Y.); guosong@scsaas.cn (S.G.); zengxiangzhong@scsaas.cn (X.Z.); zjzhou@scsaas.cn (Z.Z.); shangguan@scsaas.cn (Y.S.); mjhe9331@scsaas.cn (M.H.); ouyangyiting@scsaas.cn (Y.O.); chenkun410@scsaas.cn (K.C.)

[2] Sichuan Vegetable Engineering Technology Research Center, Chengdu 611934, China

[3] Sichuan Guojing Xingnong Investment Co., Ltd., Chengdu 610000, China; 13908181450@163.com

[4] Sichuan Institute of Edible Fungi, Chengdu 610066, China; lijingsaas@scsaas.cn

* Correspondence: qinyusheng@scsaas.cn

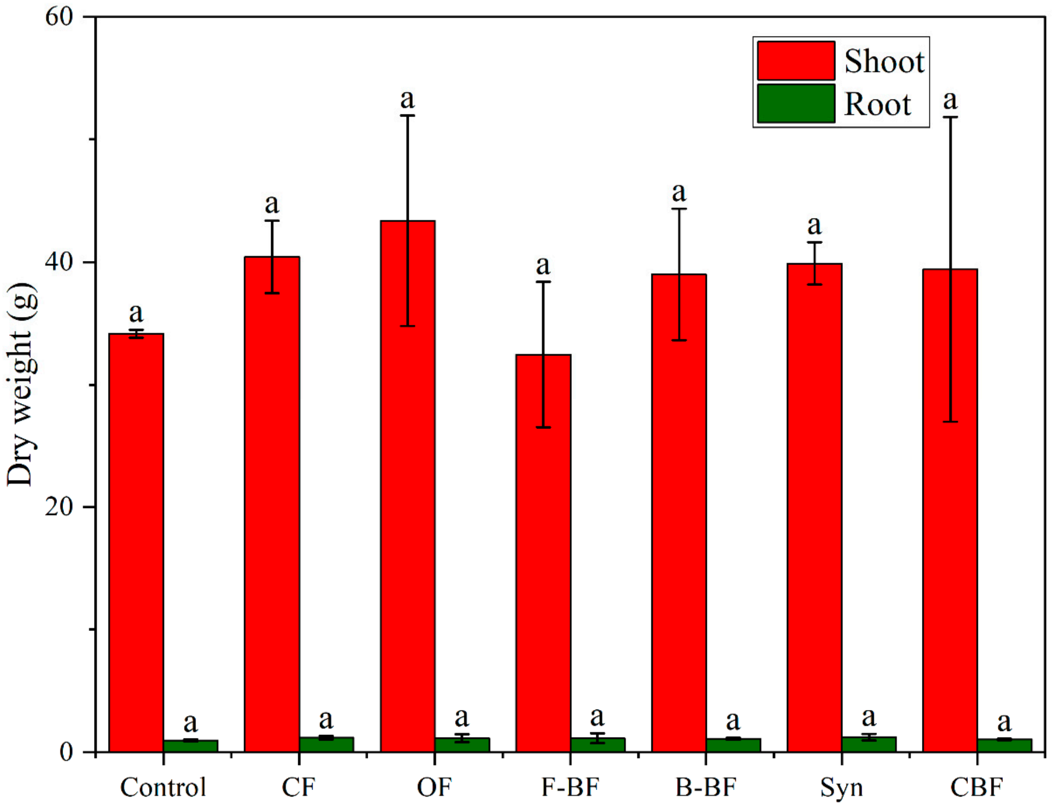

Figure S1. Dry weight of cucumber in field experiment. The field soil was naturally infested with *F. oxysporum*. Control, no fertilizer; CF, mineral fertilizers with conventional rate; OF, chemical N fertilizer reduction by 25%+7500 kg/hm^2 organic fertilizer; F-BF, chemical N fertilizer reduction by 25%+7500 kg/hm^2 fungi bio-fertilizer; B-BF, chemical N fertilizer reduction by 25%+7500 kg/hm^2 bacteria bio-fertilizer; Syn, chemical N fertilizer reduction by 25%+7500 kg/hm^2 fungi and bacteria combined bio-fertilizer; CBF, chemical fertilizer reduction by 25%+7500 kg/hm^2 commercial bio-fertilizer.

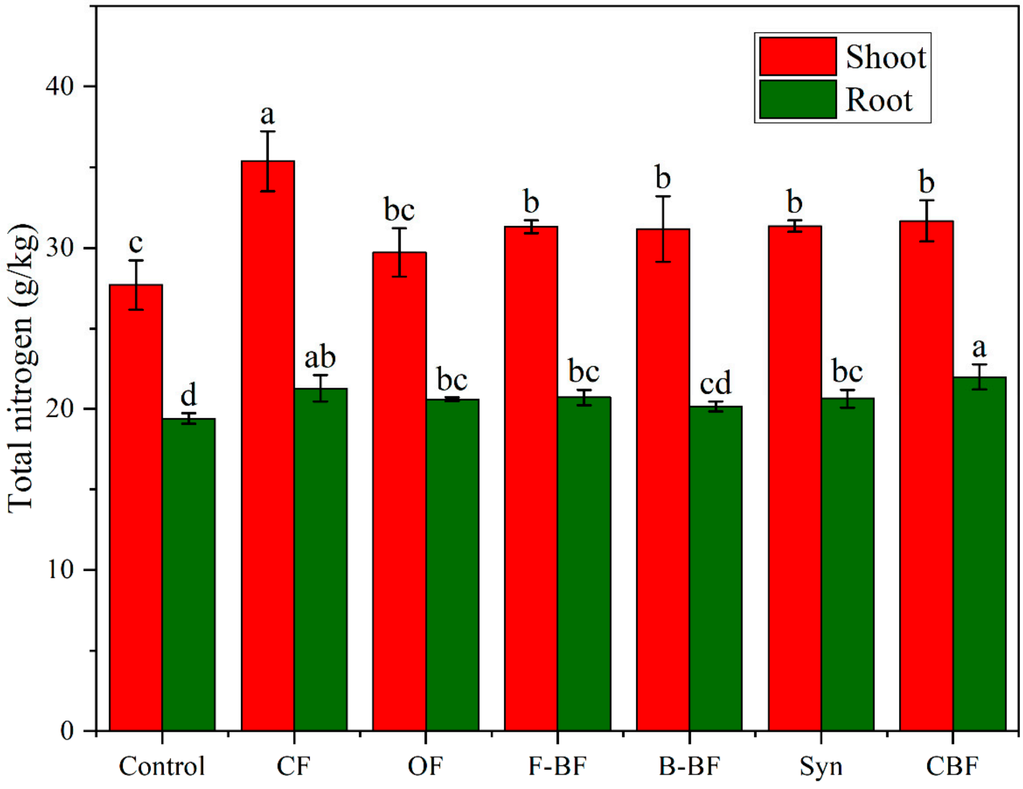

Figure S2. Total nitrogen of cucumber in field experiment. The field soil was naturally infested with *F. oxysporum*. Control, no fertilizer; CF, mineral fertilizers with conventional rate; OF, chemical N fertilizer reduction by 25%+7500 kg/hm^2 organic fertilizer; F-BF, chemical N fertilizer reduction by 25%+7500 kg/hm^2 fungi bio-fertilizer; B-BF, chemical N fertilizer reduction by 25%+7500 kg/hm^2 bacteria bio-fertilizer; Syn, chemical N fertilizer reduction by 25%+7500 kg/hm^2 fungi and bacteria combined bio-fertilizer; CBF, chemical fertilizer reduction by 25%+7500 kg/hm^2 commercial bio-fertilizer.

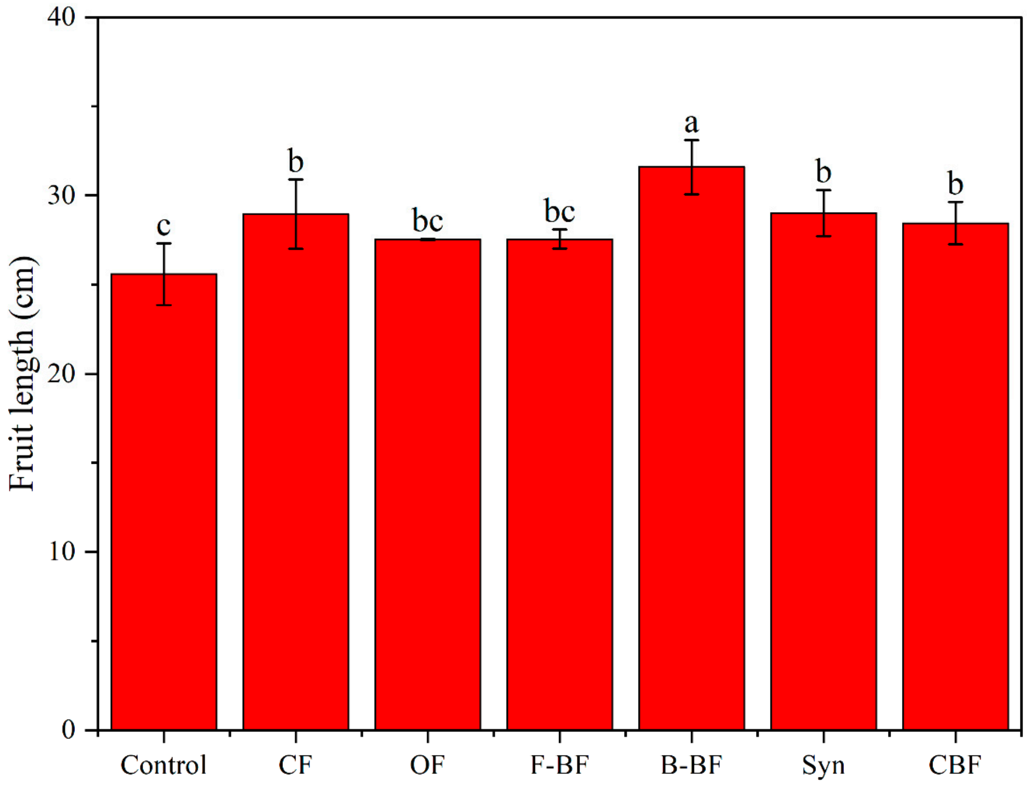

Figure S3. Fruit length of cucumber in field experiment. The field soil was naturally infested with *F. oxysporum*. Control, no fertilizer; CF, mineral fertilizers with conventional rate; OF, chemical N fertilizer reduction by 25%+7500 kg/hm^2 organic fertilizer; F-BF, chemical N fertilizer reduction by 25%+7500 kg/hm^2 fungi bio-fertilizer; B-BF, chemical N fertilizer reduction by 25%+7500 kg/hm^2 bacteria bio-fertilizer; Syn, chemical N fertilizer reduction by 25%+7500 kg/hm^2 fungi and bacteria combined bio-fertilizer; CBF, chemical fertilizer reduction by 25%+7500 kg/hm^2 commercial bio-fertilizer.

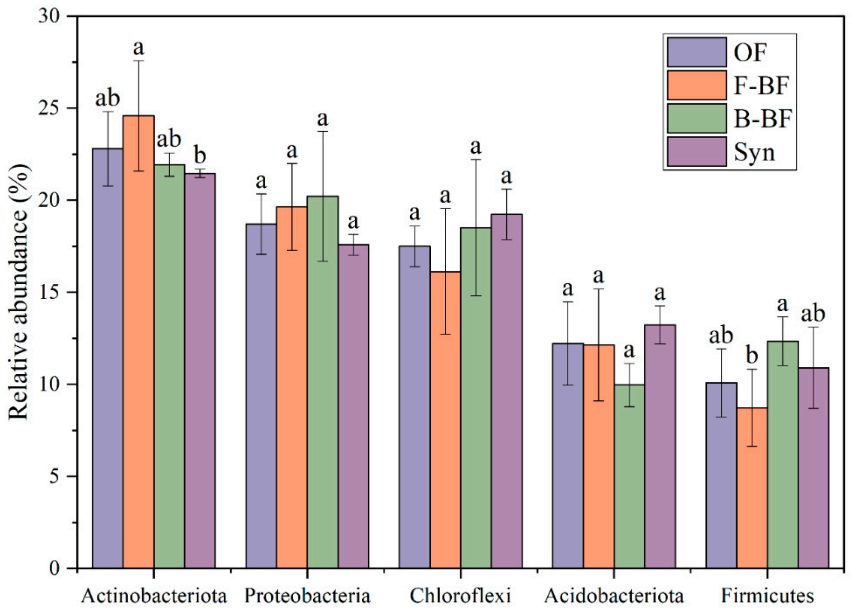

Figure S4. The relative abundance of certain bacterial phylum in cucumber rhizosphere. The field soil was naturally infested with *F. oxysporum*. OF, chemical N fertilizer reduction by 25%+7500 kg/hm^2 organic fertilizer; F-BF, chemical N fertilizer reduction by 25%+7500 kg/hm^2 fungi bio-fertilizer; B-BF, chemical N fertilizer reduction by 25%+7500 kg/hm^2 bacteria bio-fertilizer; Syn, chemical N fertilizer reduction by 25%+7500 kg/hm^2 fungi and bacteria combined bio-fertilizer.

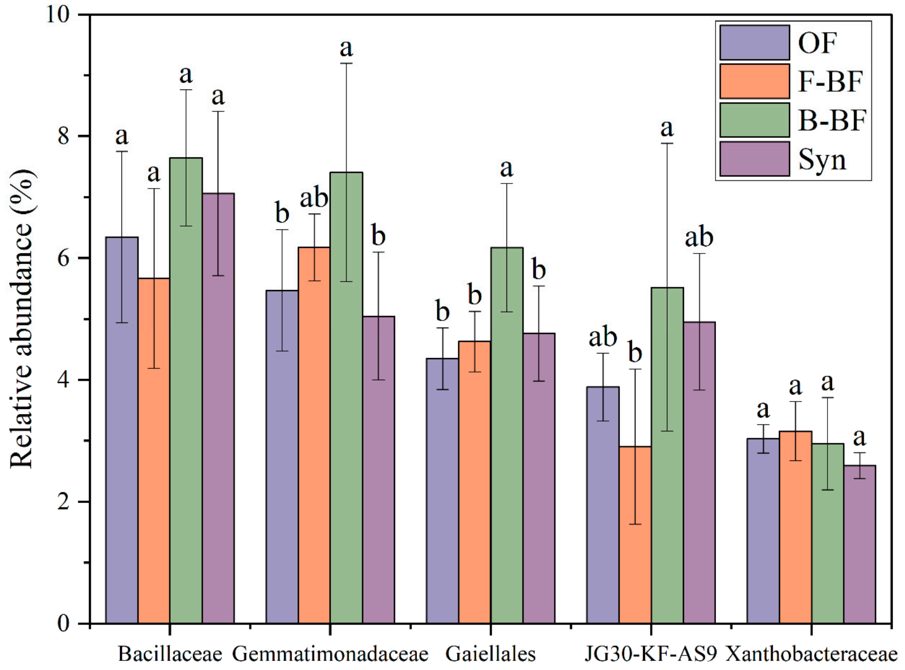

Figure S5. The relative abundance of certain bacterial family in cucumber rhizosphere. The field soil was naturally infested with *F. oxysporum*. OF, chemical N fertilizer reduction by 25%+7500 kg/hm^2 organic fertilizer; F-BF, chemical N fertilizer reduction by 25%+7500 kg/hm^2 fungi bio-fertilizer; B-BF, chemical N fertilizer reduction by 25%+7500 kg/hm^2 bacteria bio-fertilizer; Syn, chemical N fertilizer reduction by 25%+7500 kg/hm^2 fungi and bacteria combined bio-fertilizer.

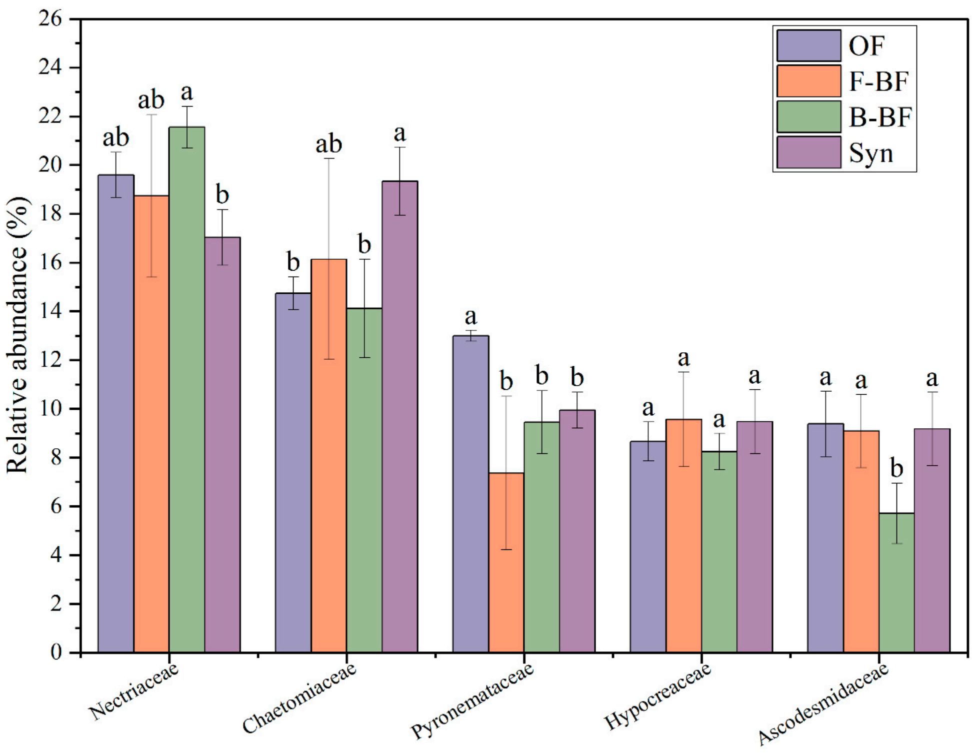

Figure S6. The relative abundance of certain fungal family in cucumber rhizosphere. The field soil was naturally infested with *F. oxysporum*. OF, chemical N fertilizer reduction by 25%+7500 kg/hm^2 organic fertilizer; F-BF, chemical N fertilizer reduction by 25%+7500 kg/hm^2 fungi bio-fertilizer; B-BF, chemical N fertilizer reduction by 25%+7500 kg/hm^2 bacteria bio-fertilizer; Syn, chemical N fertilizer reduction by 25%+7500 kg/hm^2 fungi and bacteria combined bio-fertilizer.

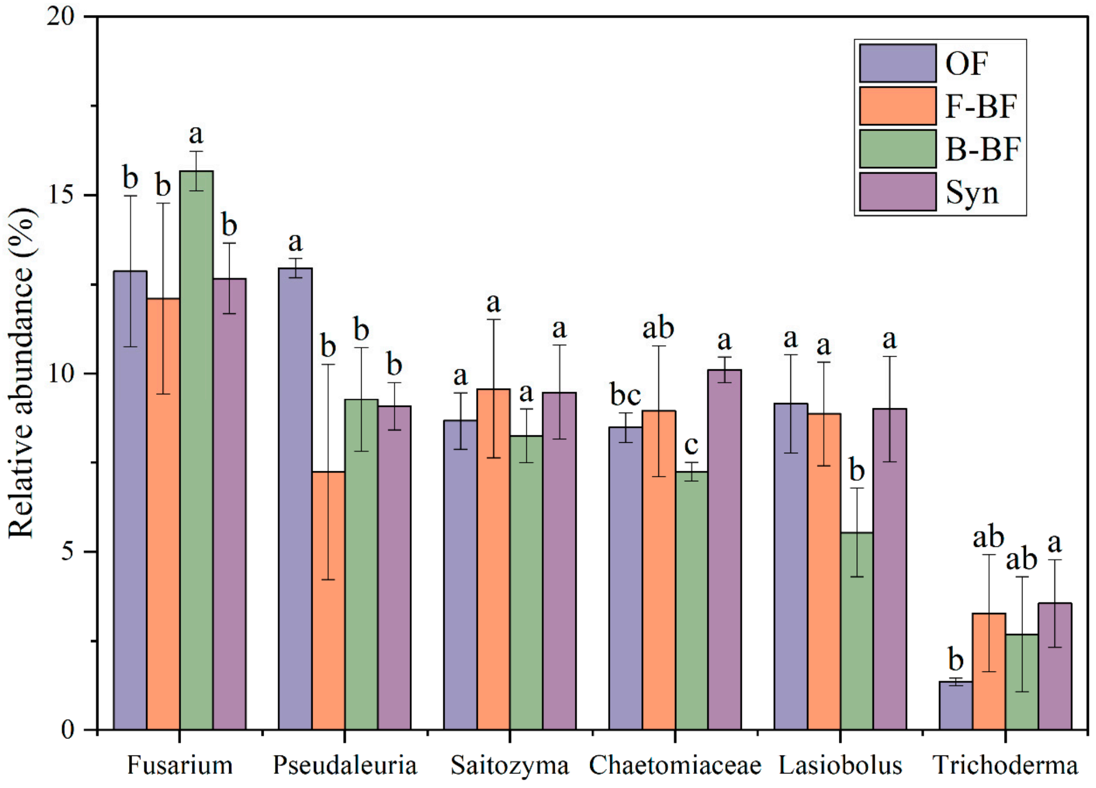

Figure S7. The relative abundance of certain fungal genus in cucumber rhizosphere. The field soil was naturally infested with *F. oxysporum*. OF, chemical N fertilizer reduction by 25%+7500 kg/hm^2 organic fertilizer; F-BF, chemical N fertilizer reduction by 25%+7500 kg/hm^2 fungi bio-fertilizer; B-BF, chemical N fertilizer reduction by 25%+7500 kg/hm^2 bacteria bio-fertilizer; Syn, chemical N fertilizer reduction by 25%+7500 kg/hm^2 fungi and bacteria combined bio-fertilizer.

Table S1. Content of amino acids of cucumber in field experiment.

Treatment	aspartic acid	Threonine	Serine	Glutamic acid	Proline	Glycine	Alanine	Glycine	Methionine	Isoleucine	Leucine	Tyrosine	Phenylalanine	Histidine	Lysine	Arginine
Control	0.0486±0.0074b	0.0250±0.0030b	0.0281±0.0039b	0.2163±0.0037e	0.0138±0.0032a	0.0291±0.0049a	0.0278±0.0052a	0.0299±0.0051b	0.0058±0.0007bc	0.0256±0.0044b	0.0407±0.0073b	0.0137±0.0023b	0.0253±0.0047b	0.0205±0.0035b	0.0347±0.0063a	0.0255±0.0035b
CF	0.054±0.0030ab	0.0280±0.0010ab	0.0320±0.0020ab	0.2600±0.0100d	0.0140±0.0010a	0.0315±0.0015a	0.0325±0.0015a	0.0335±0.0015ab	0.0055±0.0003c	0.0295±0.0015ab	0.0445±0.0025ab	0.0145±0.0015ab	0.0275±0.0015ab	0.0225±0.0005ab	0.0375±0.0025a	0.0285±0.0015ab
OF	0.0565±0.0055ab	0.0295±0.0025ab	0.0320±0.0030ab	0.2800±0.0001bc	0.0145±0.0015a	0.0320±0.0020a	0.0305±0.0035a	0.0340±0.0030ab	0.0066±0.0009ab	0.0300±0.0030ab	0.0455±0.0045ab	0.0155±0.0025ab	0.0280±0.0030ab	0.0230±0.0010ab	0.0380±0.0040a	0.0300±0.0040ab
F-BF	0.0542±0.0058ab	0.0284±0.0026ab	0.0322±0.0038ab	0.2649±0.0151cd	0.0147±0.0023a	0.0322±0.0038a	0.0313±0.0047a	0.0331±0.0039ab	0.0059±0.0001bc	0.0280±0.0030ab	0.0451±0.0059ab	0.0152±0.0018ab	0.0271±0.0029ab	0.0225±0.0025ab	0.0387±0.0053a	0.0294±0.0036ab
B-BF	0.0575±0.0015a	0.0305±0.0005a	0.0340±0.0010a	0.3100±0.0100a	0.0145±0.0005a	0.0340±0.0010a	0.0325±0.0015a	0.0355±0.0005a	0.0069±0.0002a	0.0315±0.0005a	0.0470±0.0010ab	0.0160±0.0001ab	0.0295±0.0005ab	0.0240±0.0001a	0.0395±0.0015a	0.0315±0.0015a
Syn	0.0605±0.0025a	0.0320±0.0010a	0.0345±0.0005a	0.2900±0.0100b	0.0160±0.0010a	0.0340±0.0001a	0.0330±0.0010a	0.0365±0.0015a	0.0073±0.0001a	0.0320±0.0020a	0.0490±0.0020a	0.0170±0.0010a	0.0305±0.0015a	0.0245±0.0005a	0.0415±0.0015a	0.0320±0.0010a
CBF	0.0583±0.0024a	0.0308±0.0010a	0.0332±0.0005a	0.2792±0.0096bc	0.0154±0.0010a	0.0327±0.0001a	0.0318±0.0010a	0.0351±0.0014ab	0.0070±0.0001a	0.0308±0.0019a	0.0472±0.0019ab	0.0164±0.0010ab	0.0294±0.0014ab	0.0236±0.0005ab	0.0400±0.0014a	0.0308±0.0010a

Table S2. Treatments of cucumber in field experiment.

Treatments	Nitrogen (N, kg/hm^2)	Chemical nitrogen (CN, %)	Organic nitrogen (ON, %)	Organic fertilizers (kg/hm^2)	Phosphate fertilizer (P_2O_5, kg/hm^2)	potash fertilizer (K_2O, kg/hm^2)
No fertilizer (Control)	150	75	25	7500	105	300
Chemical fertilizer (CF)	150	100	0	0	105	300
Organic fertilizer (OF)	150	75	25	7500	105	300
Fungi bio-fertilizer (F-BF)	150	75	25	7500	105	300
Bacteria bio-fertilizer (B-BF)	150	75	25	7500	105	300
Fungi and bacteria bio-fertilizer (Syn)	150	75	25	7500	105	300
Commercial bio-fertilizer (CBF)	150	75	25	7500	105	300
